# Controlling for human population stratification in rare variant association studies

**DOI:** 10.1038/s41598-021-98370-5

**Published:** 2021-09-24

**Authors:** Matthieu Bouaziz, Jimmy Mullaert, Benedetta Bigio, Yoann Seeleuthner, Jean-Laurent Casanova, Alexandre Alcais, Laurent Abel, Aurélie Cobat

**Affiliations:** 1 Laboratory of Human Genetics of Infectious Diseases, Necker Branch, INSERM U1163, Paris, France; 2 Université de Paris, Imagine Institute, 75015 Paris, France; 3 IAME, INSERM, Université de Paris, 75018 Paris, France; 4 DEBRC, AP-HP, Hôpital Bichat, 75018 Paris, France; 5 St. Giles Laboratory of Human Genetics of Infectious Diseases, Rockefeller Branch, The Rockefeller University, New York, USA; 6 Howard Hughes Medical Institute, New York, NY USA

**Keywords:** Genetic association study, Next-generation sequencing, Statistical methods

## Abstract

Population stratification is a confounder of genetic association studies. In analyses of rare variants, corrections based on principal components (PCs) and linear mixed models (LMMs) yield conflicting conclusions. Studies evaluating these approaches generally focused on limited types of structure and large sample sizes. We investigated the properties of several correction methods through a large simulation study using real exome data, and several within- and between-continent stratification scenarios. We considered different sample sizes, with situations including as few as 50 cases, to account for the analysis of rare disorders. Large samples showed that accounting for stratification was more difficult with a continental than with a worldwide structure. When considering a sample of 50 cases, an inflation of type-I-errors was observed with PCs for small numbers of controls (≤ 100), and with LMMs for large numbers of controls (≥ 1000). We also tested a novel local permutation method (LocPerm), which maintained a correct type-I-error in all situations. Powers were equivalent for all approaches pointing out that the key issue is to properly control type-I-errors. Finally, we found that power of analyses including small numbers of cases can be increased, by adding a large panel of external controls, provided an appropriate stratification correction was used.

## Introduction

Genetic association studies focusing on rare variants have become a popular approach to analyzing rare and common diseases. The advent of next-generation sequencing (NGS) and the development of new statistical approaches have rendered possible the comprehensive investigation of rare genetic variants, overcoming the shortcomings of classical genome-wide association studies (GWAS)^1,2^. When dealing with rare variants, single variant association test used in GWAS are underpowered, unless very large number of samples are available^3^. To overcome this problem, most approaches use an aggregation strategy within a genetic unit, usually a gene. These gene-based tests can be divided into two main categories: burden and variance-component tests^1,2,4,5^. Population stratification occurs when study subjects, usually cases and controls, are recruited from genetically heterogeneous populations. This problem is well known in association studies with common variants, causing an inflation of the type I error rate and reducing power. Several statistical approaches can be used to account for population stratification in GWAS. The most widely used are based on Principal Components (PC) analysis^6,7^ and Linear Mixed Models (LMM)^8–11^.

Population stratification also affects association studies including rare variants^12–14^. However, it remains unclear whether the same correction methods can be applied to rare variant association studies^13,15^, particularly as rare and common variants may induce different types of population structure^13,16^. Many studies have investigated the bias introduced by population stratification in the analysis of rare variants and have highlighted the need for corrective approaches to obtain meaningful results^13,17,18^. The performance of the correction method depends on the study setting and the method used to analyze the variants^12,13,19–22^. PC has been widely investigated^6,7,23–26^ and shown to yield satisfactory correction at large geographic scales, but not at finer scales^21^. LMM have also been studied^20,27^ and shown to account well for stratification if variance-component approaches are used to test for association^20^. Most of these studies used simulated genetic data that did not completely reproduce the complexity of real exome sequences, and limited types of population structures. In addition, they used large numbers of cases (*e.g.* generally more than 500), which may not always be possible in practice, particularly in studies focusing on rare diseases.

We aimed at addressing such limitations of classical comparative studies with the comprehensive evaluation study proposed in this article. We investigated the main correction methods for rare variant association studies in the context of the study of rare disorders for which many aspects still have to be unraveled. As a matter of fact, association studies aiming at understanding rare diseases are usually performed on small cohorts and specific genetic models. We therefore focused on limited sample sizes and modeled our disease phenotype in an appropriate manner as described hereafter. For an accurate assessment of the different approaches, we used real NGS data from two sources: 1000 Genomes data^28^ and our in-house cohort, with data for > 5000 exomes^29^. We focused on two population structure scenarios: within-continent stratification (recent separation) and between-continent stratification (ancient separation). We also considered different sample sizes, including situations with as few as 50 cases, which have, to our knowledge, never been extensively investigated in this manner. We focused on a classic genetic model for a rare disease with a phenotype driven by rare deleterious variants well suited for a burden test, such as the cohort allelic sums test (CAST)^4^. We tested two classical correction methods, PC and LMM, a promising novel correction method called adapted local permutations (LocPerm)^30^ and considered an uncorrected CAST-like test as a reference. Our global objective here is to provide useful practical insights into how best to account for population stratification in rare variant association studies.

## Materials and methods

### Simulation study

#### Exome data

For a realistic comparison of the correction approaches, we used two real exome datasets rather than program-based simulated exomes. Simulated data tend to provide erroneous site frequency spectra or LD structures^31^. The first dataset used was our HGID (Human Genetic of Infectious Diseases) database, containing 3104 samples of in-house whole-exome sequencing (WES) data generated with the SureSelect Human All Exon V4 + UTRs exome capture kit (https://agilent.com). All study participants provided written informed consent for the use of their DNA in studies aiming to identify genetic risk variants for disease. IRB approval was obtained from The Rockefeller University and Necker Hospital for Sick Children, along with a number of collaborating institutions, and all research was performed in accordance with relevant guidelines and regulations. The second dataset used was the 2504 whole genomes from 1000 Genomes phase 3 (http://www.internationalgenome.org/) reduced with the same capture kit. We merged all the exomes from these two databases into a single large dataset before selecting samples. We performed quality control, retaining only coding variants with a depth of coverage (DP) > 8, a genotype quality (GQ) > 20, a minor read ratio (MRR) > 0.2 and call-rate > 95%^32^. We then excluded all related individuals based on the kinship coefficient (King's kinship 2K > 0.1875)^33,34^, resulting in a final set of 4,887 unrelated samples. From these samples, we created two types of samples, as comparable as possible to those used in practice in association studies. The first sample, the “European” sample, consisted of samples from patients of European ancestry, and was used to assess stratification at the continental level. The second, the “Worldwide” sample, consisted of samples from European individuals together with North-African, Middle-Eastern, and South-Asian samples, for the assessment of intercontinental stratification.

#### European sample

**Figure 1:**
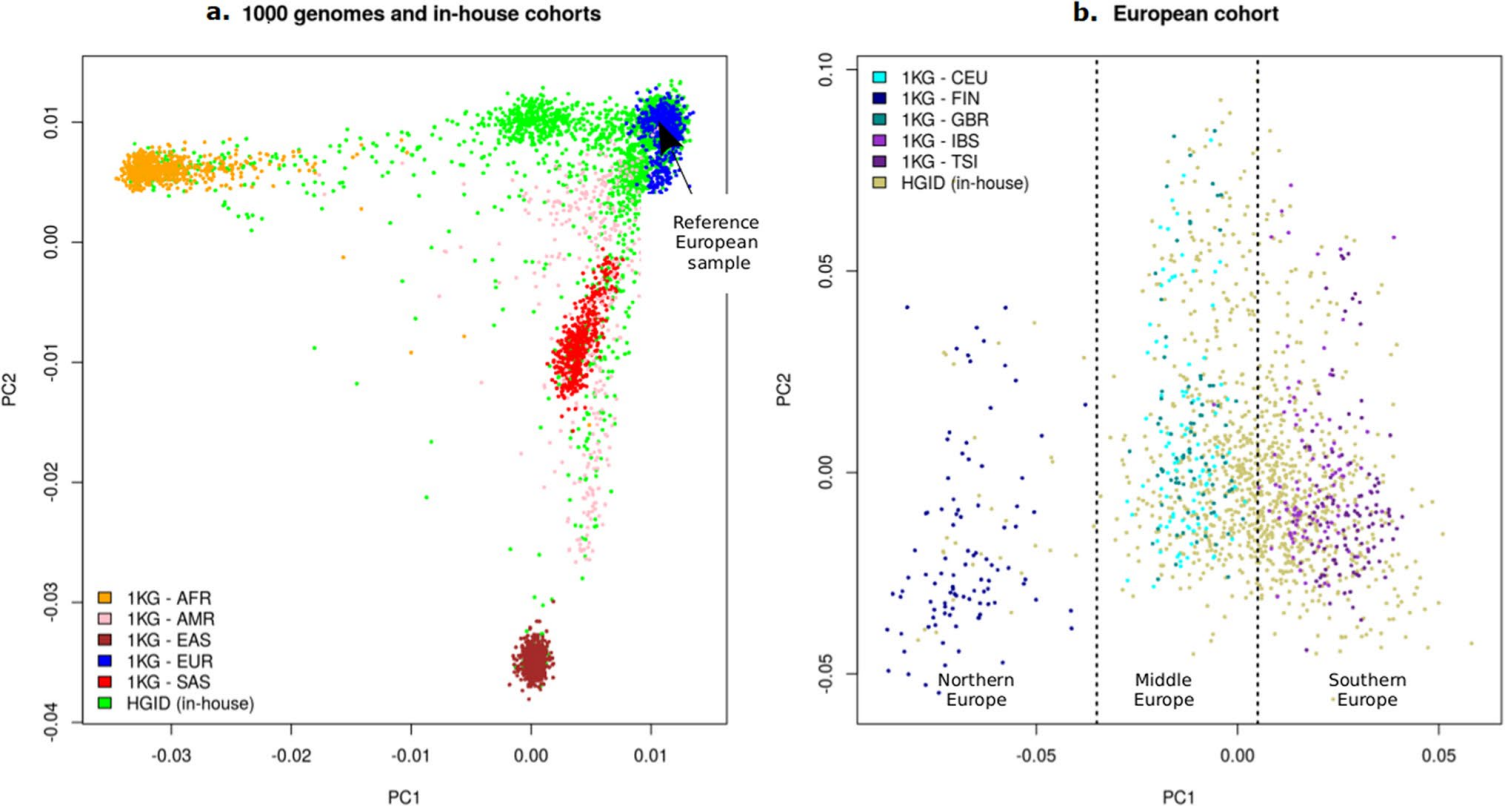
Graphical representation of the European sample. (**a**) PCA plots of the 4887 samples comprising the 3104 samples from our in-house cohort HGID and the 1000 genomes (1KG) individuals including African (AFR), Ad Mixed American (AMR), East-Asian (EAS), European (EUR) and South-Asian (SAS). Common variants were used to produce these plots. The European reference individual is singled out. (**b**) 1523 selected individuals of the final European cohort with a genetic distance to the European reference sample below the empirically derived threshold. The dashed vertical lines correspond to empirical PC1 thresholds chosen to split the samples into three European subgroups: Northern (n = 127 including 99 1KG FIN and 28 HGID samples), Middle (n = 651 including 99 1KG CEU, 91 1KG GBR and 461 HGID samples), and South European ancestry (n = 745 including 107 1KG TSI, 107 1KG IBS and 531 HGID samples. PC1 threshold were defined based on the 1000 genomes samples and further applied to HGID samples (in yellow) so that they were all assigned to one and only one subgroup for simulation purpose.

We selected samples from individuals of European ancestry based on a reference sample and a genetic distance. We first picked a European sample (sample HG00146 from the GBR population of 1000 Genomes, Fig. 1a) as a reference, based on its central position in the PCA space of the European population and calculated its genetic distance to all other samples in the combined dataset. We used a Euclidean distance based on the first 10 PCs: the distance between individuals *i* and *j* is calculated as $d_{ij}^{2} = \mathop \sum \nolimits_{k = 1}^{10} \lambda_{k} \left| {PC_{ki}^{CV} - PC_{kj}^{CV} } \right|^{2}$, where ***PC***^***CV***^ is the matrix of principal components calculated on common variants and $\lambda_{k}$ is the eigenvalue corresponding to the *k*-th principal component ${\varvec{PC}}_{{\varvec{k}}}^{{{\varvec{CV}}}}$. We considered that a sample could be “European” if its distance to the reference sample was below a certain threshold. This threshold was empirically chosen to ensure that all individuals of known European ancestry from the 1000 Genomes and our in-house HGID cohorts were included. The final sample consisted of 1523 individuals, and included all the European samples from 1000 Genomes (N = 503). As seen in Fig. 1b, this dataset included individuals with a smooth gradient of European ancestry. For simulation purposes, we empirically separated this admixed European population into three groups based on the PCA (Fig. 1b): Northern ancestry, Middle-Europe ancestry and Southern ancestry. Empirical PC1 thresholds were chosen so that all the Finnish 1000 Genomes (FIN) samples were assigned to the Northern ancestry group, all 1000 Genomes samples with Western Europe ancestry (CEU and GBR) were assigned to the Middle-Europe group and all 1000 Genomes samples with South Europe ancestry (IBR and TSI) were assigned to the Southern ancestry group. The same thresholds were further applied to the HGID samples so that they were all assigned to one of the three groups. The sample size for each subpopulation is shown in Supplementary Table S1 online. The final sample contained 328,989 biallelic SNPs and 102,219 private variants, *i.e.* variants present in only one sample (Supplementary Table S2 online).

#### Worldwide sample

**Figure 2:**
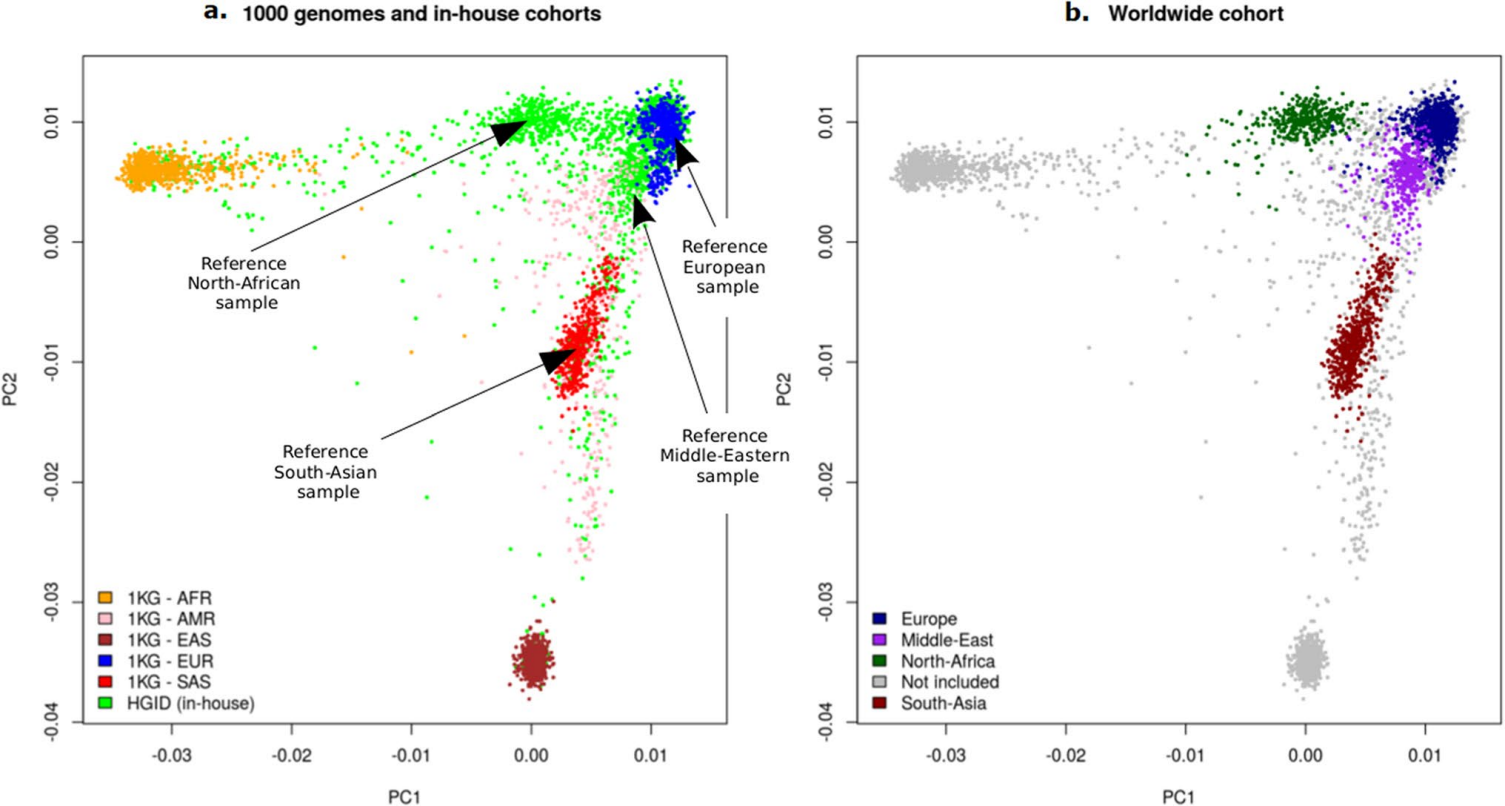
Graphical representation of the Worldwide sample. (**a**) PCA plots of the 4887 samples comprising the 3104 samples from our in-house cohort HGID and the 1000 genomes (1KG) individuals including African (AFR), Ad Mixed American (AMR), East-Asian (EAS), European (EUR) and South-Asian (SAS). Common variants were used to produce these plots. Reference individuals are singled out. These samples are then used to establish the final Worldwide cohort by considering all samples with a genetic distance to the references below given thresholds. (**b**) The selected 1,967 individuals with European (n = 700), Middle-Eastern (n = 543), North-African (n = 359) and South-Asian (n = 365) ancestries are colored. The remaining individuals are left in grey.

The Worldwide sample was created in a similar manner. We selected four different reference samples—based on their central position within the PCA space defined by each population—of European (sample HG00146 from the GBR population of 1000 Genomes), South-Asian (sample NA20847 from the GIH population of 1000 Genomes), Middle-Eastern and North-African (samples from our in-house sample with a reported and verified Middle-Eastern or North-African ancestry) ancestry (Fig. 2a). The genetic distances between each sample and the four reference samples were calculated as previously described. Thresholds on the genetic distance were empirically chosen so that each sample with a reported ancestry of interest was assigned to the corresponding population (Fig. 2b) and further applied to all the other HGID samples available. The final Worldwide sample included 1,967 individuals assigned to one of the four main ethnic groups (see Supplementary Table S1 online). Note that all the European samples of this sample were also present in the European sample. This sample contained 483,762 biallelic SNPs and 132,565 private variants (see Supplementary Table S2 online).

#### Stratification scenarios

We first assessed the various correction approaches on case/control samples with large sample sizes (*i.e.*with the whole European or Worldwide sample). We used the same three stratification scenarios for both samples. In each scenario, we considered a fixed proportion of 15% cases and 85% controls. Thus, in all our scenarios, the case/control ratio was unbalanced, as is often the case in practice. Comparison studies generally consider balanced scenarios with large numbers of cases and controls, corresponding to the ideal situation for most correction approaches, and their performance in more realistic conditions may therefore be overestimated. We considered a first scenario without stratification (No PS), in which we randomly selected 15% of the samples in each subpopulation as cases, the rest being used as controls. The second scenario corresponded to moderate stratification (Moderate PS), with the cases selected mostly from certain subpopulations. The third scenario was an extreme situation (High PS), in which all the cases were selected from a single subpopulation. The distribution of cases for the European and the Worldwide samples is shown, for each scenario, in Supplementary Table S3 online.

In practice, the samples used in rare variant association studies are frequently not very large. This is particularly true for rare diseases, for which only small numbers of cases are available. Case numbers may also be small as a consequence of the WES cost. The usual analysis strategy involves matching the controls to the cases. One key question is whether the addition of unmatched controls could increase the power of the analysis when population stratification is taken into account properly. Such controls are now available in large cohorts, such as the 1000 Genomes (Genomes Project, Auton^28^), UK10K^35^, and UK Biobank^36^ cohorts. We decided to investigate such strategies, by considering several scenarios with 50 cases and various numbers of controls of similar or different ancestries (see Supplementary Table S4 online). We considered three possible types of cases: 50 cases from the rather homogeneous Southern-Europe subpopulation (50SE), 50 cases from the more heterogeneous whole European population (50E) and 50 cases selected Worldwide (50W). Four types of controls were considered: 100 controls from the same population as the cases (100SE, 100E, 100W), 1000 controls from the total European sample (1000E), 1000 controls randomly chosen from the total Worldwide sample (1000W) and 2000 controls randomly chosen from the total Worldwide sample (2000W).

#### Type I error rate evaluation

For each type of sample and stratification scenario, the type I error rate was estimated under the null hypothesis of no association between a gene and the phenotype (*H*_0_). For large samples, we used the full European and Worldwide datasets composed of independent individuals. For each replicate, we simulated an independent vector of phenotypes according to the population structure by randomly assigning the case and control status according to the stratification proportions provided in Supplementary Table S3 and respecting a fixed proportion of cases of 15%. Each protein-coding gene was then tested for association with the phenotype by the various statistical approaches described in the Statistical methods section. The rare variants included in these tests were biallelic variants with a $MAF \leqslant 5\%$ in the sample analyzed (including private variants). We included only genes with at least 10 rare variant carriers in the considered dataset, resulting in 17,619 genes being studied in the European sample, and 17,854 genes in the Worldwide sample. For small sample size, a similar simulation process was applied and samples were drawn without replacement from the main cohorts, so that there were not any duplicated individuals in any given replicate, according to the proportions of cases and controls in the different populations described in Supplementary Table S4 online. In these scenarios, the number of genes with at least 10 mutation carriers retained depended on sample size (see Supplementary Table S5 online). For each scenario, we generated 10 replicates to account for sampling variation. The type I error rate at the nominal level $\alpha$ was evaluated by assessing the quantity $fp = \frac{{\# \left\{ {p{ - }value_{i} \leqslant \alpha ,\quad i = 1, \ldots ,G} \right\}}}{G}$ where *G* is the total number of genes tested. We decided to provide an adjusted prediction interval (PI), accounting for the number of methods investigated, with the type I error rate as suggested in previous studies^20^. The bounds of this interval are $fp \pm Z_{{0.975/\# \left( {methods} \right)}} \sqrt {fp\left( {1 - fp} \right){\text{/G}}}$ where $Z_{{0.975/\# \left( {methods} \right)}}$ replaces the usual 97.5 percentile of the normal distribution $Z_{0.975}$ after adjustment for the number of methods investigated. An approach was considered to provide a good correction if its type I error rate was found within this interval.

#### Power studies

Power was estimated under the alternative hypothesis of an association between a gene and the phenotype (*H*_1_). We selected a subset of 10 genes for the power analysis. All these genes had a cumulative frequency of rare variants (*i.e.*with $MAF \leqslant 5\%$) of ~ 10% (*i.e.* ~ 20% of carriers) and at least 10 mutation carriers. In addition, we considered ~ 50% of the rare variants of each gene to be causal, with the same direction of effect, and used the presence of at least one of these variants to define the binary genetic score described in the Statistical method section. This implies that there was no cumulative effect of carrying several causal variants, and that the relative risk is defined at the gene level. Supplementary Table S6 online provides details of the 10 genes selected and their causal variants for the European and Worldwide samples. For each sample we simulated a phenotype based on the binary genetic score and the corresponding penetrance. Penetrance for carriers and non-carriers was computed separately for each ethnic subgroup and each population stratification scenario from the observed frequency of carriers of at least one causal variant, the frequency of the disease (which varies according to the level of population stratification) and the relative risk (RR = {1, 2, 3, 4}). A detailed example of the penetrance estimation is presented in Supplementary Table S7 online for the first gene tested. We performed 500 independent replicates per gene. Power was estimated by evaluating the same quantity as for the type I error rate averaged over the 10 genes and the 500 replicates.

### Statistical methods

#### Association test

Let us now consider an association study including *n* individuals. The binary phenotype is denoted ***Y*** = (*y*_1_, …, *y*_*n*_), where *y*_*i*_ is the status of individual *i* coded 0 (healthy) or 1 (affected). We call ***X*** = (*x*_*ij*_)_*i*=1…*n*, *j*=1…*p*_ the *n* × *p* genotype matrix for *n* individuals and *p* markers. Each term *x*_*ij*_ corresponds to the genotype of sample *i* at marker *j* and is coded 0, 1 or 2 according to the number of minor alleles. We also introduce the normalized genotype matrix ${\tilde{\mathbf{X}}}$ = $\left( {\tilde{x}_{ij} } \right)_{i = 1 \ldots n, \, j = 1 \ldots p}$, where each term is $\tilde{x}_{ij} = \frac{{x_{ij} - \mu_{j} }}{{\sqrt {f_{j} \left( {1 - f_{j} } \right)} }}$ with $\mu_{j}$ the column mean and $f_{j}$ the observed allele frequency of each marker.

Several routine statistical tests are available for assessing the association between rare variants and a phenotype^4,5,37–39^. Considering our focus on a small number of cases with phenotypes driven by the presence of at least one causal variant, the most appropriate approach is that based on the CAST method^4^. This approach collapses variants into a single genetic score that takes a value of 0 if there are no rare variants in the region or 1 if there is at least one variant. Considering a given genetic region *g*, in our case a gene, the score for this region is denoted ***Z***_***g***_ = (*z*_g1_, …, *z*_*gn*_), where *z*_*gi*_ = *I* (at least one rare variant in the region *g* for individual *i*), *I*() being the indicator function.

The corresponding association test can be expressed in a logistic regression framework.

1 $$logit\left( {P\left( {{\varvec{Y}} = 1} \right)} \right) = \alpha + \beta_{g} {\varvec{Z}}_{{\varvec{g}}}$$

where $\alpha$ and $\beta_{g}$ are the model parameters for the intercept and the genetic score. Under the null hypothesis of no association $\left\{ {\beta_{g} = 0} \right\}$ the likelihood ratio test (LRT) statistics follow a $\chi_{1df}^{2}$ distribution.

For some scenarios, we also considered a quantitative genetic score in the logistic regression framework where *z*_*gi*_ is the weighted sum of the number of minor alleles with the weight for each variant being a function of its minor allele frequency. For a variant k, we defined a weight $w_{k} = \frac{1}{{\sqrt {MAF_{k} \left( {1 - MAF_{k} } \right)} }}$, as proposed by Madsen Browning^38^ for the weighted sum statistic (WSS) and denoted this approach WSS. We also considered the variance-component “sequence kernel associated test” (SKAT)^5^, as implemented in the R package SKAT.

#### Genetic similarity

Certain methods, including PC and LMM, account for population stratification by using a large number of single-nucleotide polymorphisms (SNPs) to derive genetic similarity matrices (also called relatedness matrices). Considering a set *H* of *p*_*H*_ SNPs, a normalized similarity matrix ${\varvec{S}}^{{\varvec{H}}} = \tilde{\user2{X}}^{{\varvec{H}}} \tilde{\user2{X}}^{{\user2{H^{\prime}}}}$ can be derived, where $\tilde{\user2{X}}^{{\varvec{H}}}$ is the normalized genotype matrix reduced to the markers of set *H*. Each term *s*_*ik, i*=1…*n*, *k*=1…*n*_ represents the genetic similarity between samples *i* and *k* based on the SNPs of set *H*.

With whole-exome sequencing data, a broad range of SNPs are now available, and it is usual to separate them into categories based on their minor allele frequencies (MAFs)^19,20,25^. We will consider four categories of variants, based on the MAFs calculated for the total sample: rare variants (RVs; $0\% < MAF < 1\%$), low-frequency variants (LFVs; $1\% \leqslant MAF < 5\%$), common variants (CVs; $MAF \geqslant 5\%$) and all variants (ALLVs; the union of RVs, LFVs and CVs). We excluded private variants from these sets of variants, because their sparse distribution tends to have a strong influence on the calculation of similarity matrices (Note that private variants are only excluded from the Genetic similarity matrices calculations but are included in the association testing phase). We also pruned all these sets to remove variants with a pairwise r^2^ > 0.2, to reduce the effect of linkage disequilibrium. We investigated the effect of using these different sets of SNPs $H \in \left\{ {RVs,LFVs,CVs,ALLVs} \right\}$ to derive PC-based or LMM corrections.

#### Principal component (PC) approach

PC analysis creates new variables from SNP data, the principal components, corresponding to axes of genetic variation. These variables can be included, as covariates, in a statistical model, such as the one described above to adjust for population stratification. Principal components ${\varvec{PC}}^{{\varvec{H}}} = \left( {PC_{1}^{H} , \ldots ,PC_{n - 1}^{H} } \right)$ are based on a given set of SNPs *H* and are derived from the singular vector decomposition of the normalized similarity matrix ***S***^***H***^. After adjustment for the first *m* principal components, the corresponding logistic model becomes:

2 $$logit\left( {P\left( {{\varvec{Y}} = 1} \right)} \right) = \alpha + \beta_{g} {\varvec{Z}}_{{\varvec{g}}} + \gamma_{1} {\varvec{PC}}_{1}^{{\varvec{H}}} + \cdots + \gamma_{m} {\varvec{PC}}_{{\varvec{m}}}^{{\varvec{H}}}$$

where $\gamma_{1} , \ldots ,\gamma_{m}$ are new model parameters for the PCs.

Under the null hypothesis of no association $\left\{ {\beta_{g} = 0} \right\}$, the LRT statistics follow a $\chi_{1df}^{2}$ distribution. We investigated correction based on the first 3, 5, 10 or 50 PCs, calculated on the four possible sets of variants, RVs, CVs, LFVs and ALLVs. In the following, we use a notation such that PC3_CV_, for example, indicates that the first three PCs based on common variants were used.

#### Linear mixed models (LMM)

Linear mixed models were initially developed to alleviate the effect of familial relatedness in association analyses, and have also been used to correct for population stratification in GWAS. This regressive approach considers both fixed and random effects and uses a genetic similarity matrix to improve estimation of the parameters of interest. Using the previous CAST regression framework, the LMM model becomes:

3 $${\varvec{Y}} = \alpha + \beta_{g} {\varvec{Z}}_{{\varvec{g}}} + {\varvec{u}} + \in$$

where ${\varvec{u}} \sim MVN\left( {0,\tau {\varvec{S}}^{{\varvec{H}}} } \right)$ is a vector of random effects based on the similarity matrix ***S***^***H***^ and an additional variance parameter $\tau$. Under the null hypothesis of no association $\left\{ {\beta_{g} = 0} \right\}$, the LRT statistics follow a $\chi_{1df}^{2}$ distribution. We focus here on LMM based on the relatedness matrices constructed with the four sets of variants previously described, and with for instance the notation LMM_CV_ indicating that common variants were used.

#### Adapted local permutations (LocPerm)

Permutation strategies have been designed to derive *p-*values when the 'true' null distribution of the test statistic *T*_0_ is unknown^40^. This is the case for population stratification, which creates a bias that cannot be numerically derived. The rationale behind permutation procedures is to simulate several test statistics (*T*_1_, …, *T*_*B*_) under the null hypothesis, to derive an approximated distribution as close as possible to the unknown true null distribution, and to use these statistics to estimate a *p-*value. With the classical permutation approach, the simulation of test statistics under *H*_0_ is achieved by randomly resampling phenotypes (*i.e.* exchanging them between individuals). Adapted local permutations are based on the observation that, in the presence of population structure, not all phenotypes are exchangeable^30^. However, this assumption is no longer valid in the presence of population structure. A given sample has a higher chance of sharing its phenotype with another sample of the same ancestry. Few approaches have been proposed for handling confounding factors such as population stratification in permutations. Epstein and colleagues^41^ proposed to estimate the odds of disease conditional on covariates under a null model of no genetic association and to resample individual phenotypes using these conditional disease probabilities as individual weights to obtain permuted data with a similar PS. However, subsequent studies showed that this procedure was less efficient than regular PC correction for dealing with fine-scale population structure^21^. Recently, a general new method, the *conditional permutation test*, for testing the conditional independence of variables X and Y given a potentially high dimensional random vector Z that may contain confounding factors was proposed^42^. The test permutes entries of X non-uniformly, to respect the existing dependence between X and Z and thus to account for the presence of these confounders. However, like the conditional randomization test of Candès et al.^43^, the test relies on the availability of an approximation to the distribution of X/Z and sensitivity analysis to the misspecification of the distribution parameters showed that the method suffered from a type I error inflation increasing with the misspecification level^42^.

More recently, we proposed a simpler method based on adapted local permutations. The principle is to establish, for each sample, a neighborhood, *i.e.* a set of relatively close samples with whom it is reasonable to exchange phenotypes. Each sample neighborhood is based on a genetic distance derived from the sample coordinates along the first 10 principal component axes calculated on common variants:

4 $$d_{ij}^{2} = \mathop \sum \limits_{k = 1}^{10} \lambda_{k} \left| {PC_{ki}^{CV} - PC_{kj}^{CV} } \right|^{2}$$

where ***PC***^***CV***^ is the matrix of principal components calculated on the set of common variants and $\lambda_{k}$ is the eigenvalue corresponding to the *k*-th principal component ${\varvec{PC}}_{{\varvec{k}}}^{{{\varvec{CV}}}}$. Note that a number of 10 PCs was selected since we observed, in many datasets, that the proportion of variance explained was high and the inclusion of other PCs did not have a significant impact on the resulting distances. One can then set a number *N* and select permutations that ensure that each phenotype is drawn from the *N* nearest neighbors. Such permutations are called local permutations and are selected with a Markov chain Monte Carlo sampler described in^30^. We focus on a number of *N* = *30* neighbors as it has been proposed to be a reasonable choice from a sensitivity analysis described in^30^. Permutations can then be performed for each sample, within its neighborhood.

A straightforward empirical way to derive a *p-*value for the permutation test is to assess the quantity $pv = \# \left\{ {T_{i} \geqslant T_{0} } \right\}/B$ where # is the cardinal function and *B* is the number of permutations. This method is dependent on the number of permutations computed, and a large number of permutations is required for the accurate estimation of small *p*-values. Mullaert et al. proposed an alternative semi-empirical approach, in which a limited number of resampled statistics are used to estimate the mean (m) and standard deviation (σ) of the test statistic under *H*_0_. The previously described CAST-like LRT statistics are used, through their square roots with a sign attributed according to the direction of the effect, $T_{i} = sign\left( {effect} \right)\sqrt {\left| {LRT} \right|}$, to estimate the N(m, σ^2^) distribution parameters and then calculate the *p-*value*.* We evaluated both the semi-empirical approach using 500 local permutations and the full-empirical approach using 5000 local permutations. These two approaches yielded very similar results. We therefore present here only the results for the semi-empirical approach.

#### Implementation of the simulations and methods

We used R software (https://www.R-project.org/) to code the comparison pipeline and implement the logistic CAST, logistic WSS and permutation models. The SKAT test used was implemented in the SKAT R package. Principal components and similarity matrices were obtained with Plink2 software (https://www.cog-genomics.org/plink/2.0/), and GEMMA was used for the LMM method^11,44^.

## Results

### Large sample size study

The results of the simulation study under the null hypothesis for the European sample of 1,523 individuals are presented in Table 1 (for α = 0.001) and Supplementary Table S8 (for α = 0.01). In the absence of stratification, the four methods had correct type I error rates, within the 95% PI bounds (Table 1A, Supplementary Table S8 online). This was the case for PC3 and LMM, regardless of the type of variant considered. In the presence of moderate stratification (Table 1B, Supplementary Table S8 online), the unadjusted CAST approach displayed the expected inflation of type I error rate (0.00163 at α = 0.001). The PC3 method corrected properly regardless of the type of variant at α = 0.001, but a slight inflation of type I error was observed for RVs and LFVs at α = 0.01. The use of LMM led to an inflation of type I error rates at α = 0.001, unless all variants were considered, while it gave rates within the 95% PI at α = 0.01. LocPerm had a correct type I error rate at both α levels. In the presence of strong stratification (Table 1C, Supplementary Table S8 online), the unadjusted CAST method gave a strong inflation of type I error rate, to 0.00359 at α = 0.001. The PC and LMM approaches also led to inflated type I errors (between 0.00133 and 0.00175 at α = 0.001), the lowest level of inflation being observed when CVs or all variants were considered. Thus, in the presence of strong population structure, classical methods were unable to handle the stratification properly. The adapted local permutations approach was the only method able to correct for stratification in this scenario, with a slightly conservative result of 0.00863 at α = 0.01 (Supplementary Table S8 online).

**Table 1:** Type I error rates of the different approaches for the large European sample.

	CAST	PC3	LMM	LocPerm
**A—No stratification**				
RVs	0.00106	0.00108	0.00118	0.00082
LFVs		0.0011	0.00119	
CVs		0.00104	0.00118	
ALLVs		0.00108	0.00116	
**B—Moderate stratification**				
RVs	**0.00163**	0.00117	**0.00141**	0.00095
LFVs		0.00101	**0.00125**	
CVs		0.001	**0.00124**	
ALLVs		0.00102	0.00117	
**C—High stratification**				
RVs	**0.00359**	**0.00157**	**0.00175**	0.00087
LFVs		**0.00137**	**0.00176**	
CVs		**0.00136**	**0.00161**	
ALLVs		**0.00133**	**0.00145**	

The nominal level alpha considered is $\alpha = 0.001$ and the corresponding 95%PI adjusted for the 10 methods is [0.00079–0.00121]. Type I error rates under the lower bound of the 95%PI are displayed in italic and above the upper bound of the 95%PI in bold.

We further investigated the impact of the PC correction on type I errors rates in the European sample when increasing the number of PCs and for alternative association methods (i.e. WSS and SKAT). With CAST, increasing the number of PCs did not improve the correction, a result consistent with previous findings reported by Persyn et al. (2018). The use of 50 PCs resulted in an inflation of type I error rates whatever the level of stratification, probably due to an over-adjustment of the regression model (see Supplementary Table S9 online). Thus, we used 3 PCs correction in further analyses. Under no stratification, WSS and SKAT, with or without adjustment on the first 3 PCs, had correct type I error rates at α = 0.001 (Supplementary Table S10 online). In the presence of moderate or high stratification, type I error rates of WSS and SKAT were inflated (0.0018 and 0.0024 at α = 0.001 in the presence of moderate inflation) with larger inflation observed for SKAT. Note that both WSS and SKAT had more inflated type I errors than CAST, consistent with previous reports^18^. PC3 correction method reduced the type I error inflation of WSS and SKAT under moderate and high stratification providing type I error rates close to the expected nominal level despite a failure to fully correct the type I error of WSS under the high stratification scenario (0.0017 at α = 0.001) and of SKAT under both moderate and high stratification scenario (0.00127 and 0.00129, respectively, at α = 0.001). Overall, PC3 correction performed similarly for WSS and SKAT, as compared with CAST, and we focused on CAST for subsequent analyses.

The results of the simulation study under *H*_0_ for the Worldwide sample of 1967 individuals are presented in Table 2 (for α = 0.001) and Supplementary Table S11 (for α = 0.01). In the absence of stratification, none of the main approaches had a significantly inflated type I error rate (Table 2A and Supplementary Table S11 online). At $\alpha$ = 0.01, LMM corrections were slightly conservative. The presence of moderate or strong stratification led to extremely inflated type I errors at α = 0.001 for the unadjusted CAST approach, with values of 0.00681 and 0.137, respectively. For PC3 and LMM, a satisfactory correction was obtained at α = 0.001 with CVs, whereas, at α = 0.01, PC gave a slight inflation of type I error and LMM results were slightly conservative. The three other types of variants could not properly account for stratification for PC3 and LMM. Increasing the number of PCs did not improve the results obtained with PC3 (Supplementary Table S12) for the Worldwide sample. LocPerm maintained a correct type I error rate in both scenarios, with values of 0.00096 and 0.00113 at α = 0.001 for moderate and strong stratification, respectively. Overall, the analyses under the null hypothesis within the European and Worldwide samples showed that accounting for stratification was generally more difficult with a continental structure than with a worldwide structure. PC3 and LMM based on CVs were capable of maintaining a correct type I error rate in most of the situations considered, with the exception of high levels of stratification in Europe, and LocPerm correctly accounted for stratification in all the situations considered.

**Table 2:** Type I error rates of the different approaches for the large Worldwide sample.

	CAST	PC3	LMM	LocPerm
**A—No stratification**				
RVs	0.00085	0.00099	0.00093	0.00087
LFVs		0.00099	0.00094	
CVs		0.00099	0.00093	
ALLVs		0.00099	0.00093	
**B—Moderate stratification**				
RVs	**0.00681**	**0.00259**	**0.00456**	0.00096
LFVs		0.00109	**0.00123**	
CVs		0.00105	0.00117	
ALLVs		**0.00128**	**0.00162**	
**C—High stratification**				
RVs	**0.13698**	**0.00662**	**0.01834**	0.00113
LFVs		0.0012	**0.00163**	
CVs		0.00119	0.00115	
ALLVs		**0.00127**	**0.00266**	

The nominal level alpha considered is $\alpha = 0.001$ and the corresponding 95%PI adjusted for the 10 methods is [0.00079–0.00121]. Type I error rates under the lower bound of the 95%PI are displayed in italic and above the upper bound of the 95%PI in bold.

**Figure 3:**
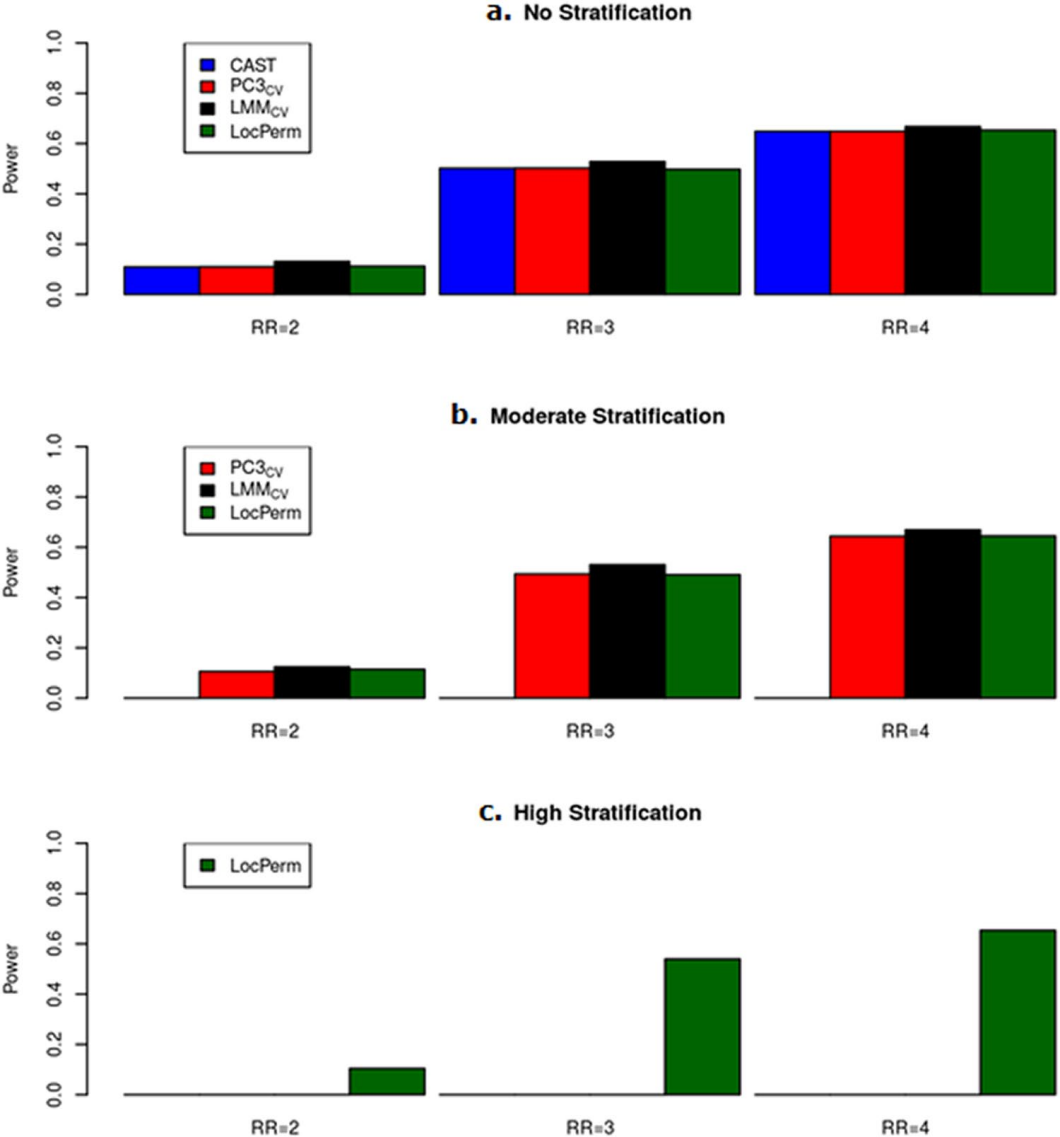
Histogram of powers for methods with a correct type I error rate for the large size European sample (n = 1523) at the level $\alpha = 0.001$. (**a**) Without stratification. (**b**) With moderate stratification. (**c**) With high stratification. Relative risks considered vary from 2 to 4 on the x-axis.

**Figure 4:**
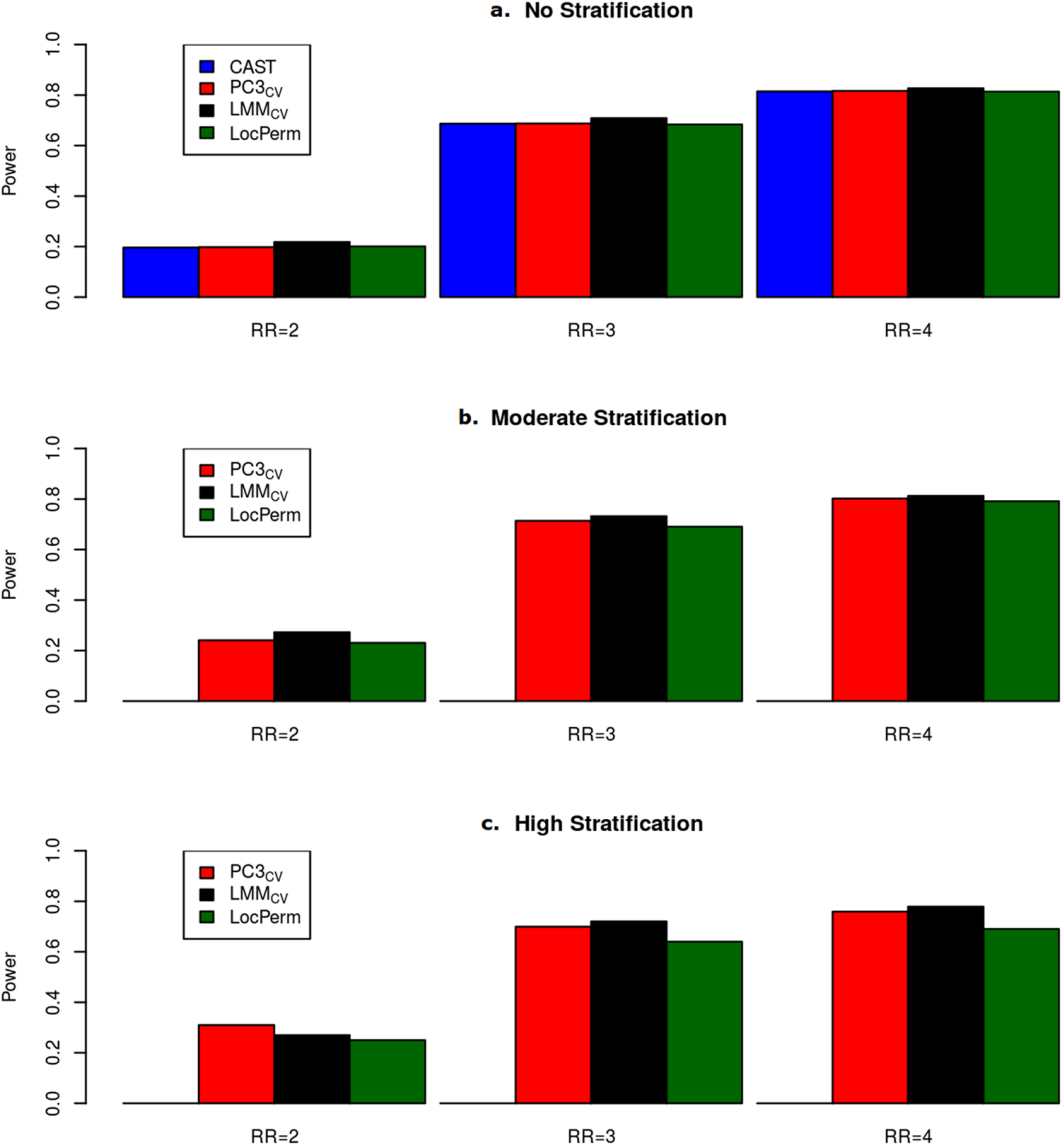
Histogram of powers for methods with a correct type I error rate for the large size Worldwide sample (n = 1967) at the level $\alpha = 0.001$. (**a**) Without stratification. (**b**) With moderate stratification. (**c**) With high stratification. Relative risks considered vary from 2 to 4 on the x-axis.

With respect to the results of the simulation under *H*_0_, we focused the power studies on the methods providing satisfactory correction (*i.e.* PC3_CV_, LMM_CV_ and LocPerm), in addition to the unadjusted CAST. Only powers derived from a correct type I error rate under *H*_0_ are presented in the main text. Adjusted powers accounting for inflated type I error rates are provided in the Supplementary figures online for information. The results of the power study for the European sample are presented in Fig. 3 and Supplementary Fig. S1 online. In situations with no stratification or moderate stratification, all approaches had similar powers, of about 50% at α = 0.001 for a relative risk of 3, for example (Fig. 3). In the presence of strong stratification, only LocPerm was able to correct for confounding and to maintain power levels (Fig. 3c). The adjusted powers (Supplementary Fig. S1 online) indicate that all three correction methods provide very similar powers when type I error is controlled. The results of the power study for the Worldwide sample are presented in Fig. 4 and Supplementary Fig. S2. As for the European sample, all methods had similar powers in the absence of stratification or the presence of moderate stratification. In the presence of strong stratification, LocPerm was slightly less powerful than the other methods (Fig. 4c) with for a RR of 3 at α = 0.001, a power of 64% as opposed to the powers of 69 and 72% obtained for PC3_CV_, and LMM_CV_, respectively. It is also interesting to compare the power of each method, separately, between the different stratification scenarios (Supplementary Fig. S3 for the European sample and Supplementary Fig. S4 for the Worldwide sample). Power was very similar for any given technique in the different stratification scenarios, indicating that the correction methods maintained the level of power observed in the absence of stratification.

### Small sample size study

The results of the simulation study under the null hypothesis for a small sample size, based on 50 cases, are presented in Table 3 (for α = 0.001) and Supplementary Table S13 online (for α = 0.01). Only PC3_CV_, LMM_CV_ and LocPerm, which provided a satisfactory correction for stratification in the large sample study, were investigated for small sample sizes. In scenarios without stratification (i.e. controls and cases of the same origin), an inflation of type I errors was observed: 1) with PC3 (about 0.0015 at α = 0.001) when the number of controls was low (100), and, to a lesser extent, with CAST (about 0.0012 at α = 0.001), and 2) with LMM (about 0.002 at α = 0.001) when the number of controls was high (1000 or 2000). In the presence of stratification (*i.e.* a large number of controls with an origin different from that of the cases), a strong inflation of type I error rates was observed for CAST. This was also the case for LMM_CV_, albeit to a lesser extent, particularly for stratification within Europe or when the cases came from the Worldwide sample and the controls from Europe only. Both PC3_CV_ and LocPerm provided correct type I error rates in all the scenarios considered with small numbers of cases and a large number of controls.

**Table 3:** Type I error rates of the different approaches for the small sample scenarios.

Scenario	CAST	PC3_CV_	LMM_CV_	LocPerm
50SE-100SE	0.0012	**0.0015**	0.0012	0.0009
50SE-1000E	**0.0016**	0.0012	**0.0028**	0.0008
50SE-1000W	**0.0046**	0.0011	**0.0015**	0.0010
50SE-2000W	**0.0046**	0.0010	**0.0016**	0.0011
50E-100E	**0.0014**	**0.0015**	0.0012	0.0010
50E-1000E	0.0010	0.0010	**0.0021**	0.0009
50E-1000W	**0.0051**	0.001	**0.0014**	0.0010
50E-2000W	**0.0050**	0.0009	**0.0015**	0.0011
50World-100W	**0.0013**	**0.0015**	0.0012	0.0010
50World-1000E	**0.0077**	*0.0007*	**0.0053**	0.0010
50World-1000W	0.0009	0.0010	**0.0021**	0.0009
50World-2000W	0.0009	0.0009	**0.002**	0.0010

The nominal level alpha considered is $\alpha = 0.001$. Type I error rates under the lower bound of the 95%PI are displayed in italic and above the upper bound of the 95%PI in bold.

Supplementary Table S3 provides the adjusted 95%PI for the different number of genes tested in each scenario.

**Figure 5:**
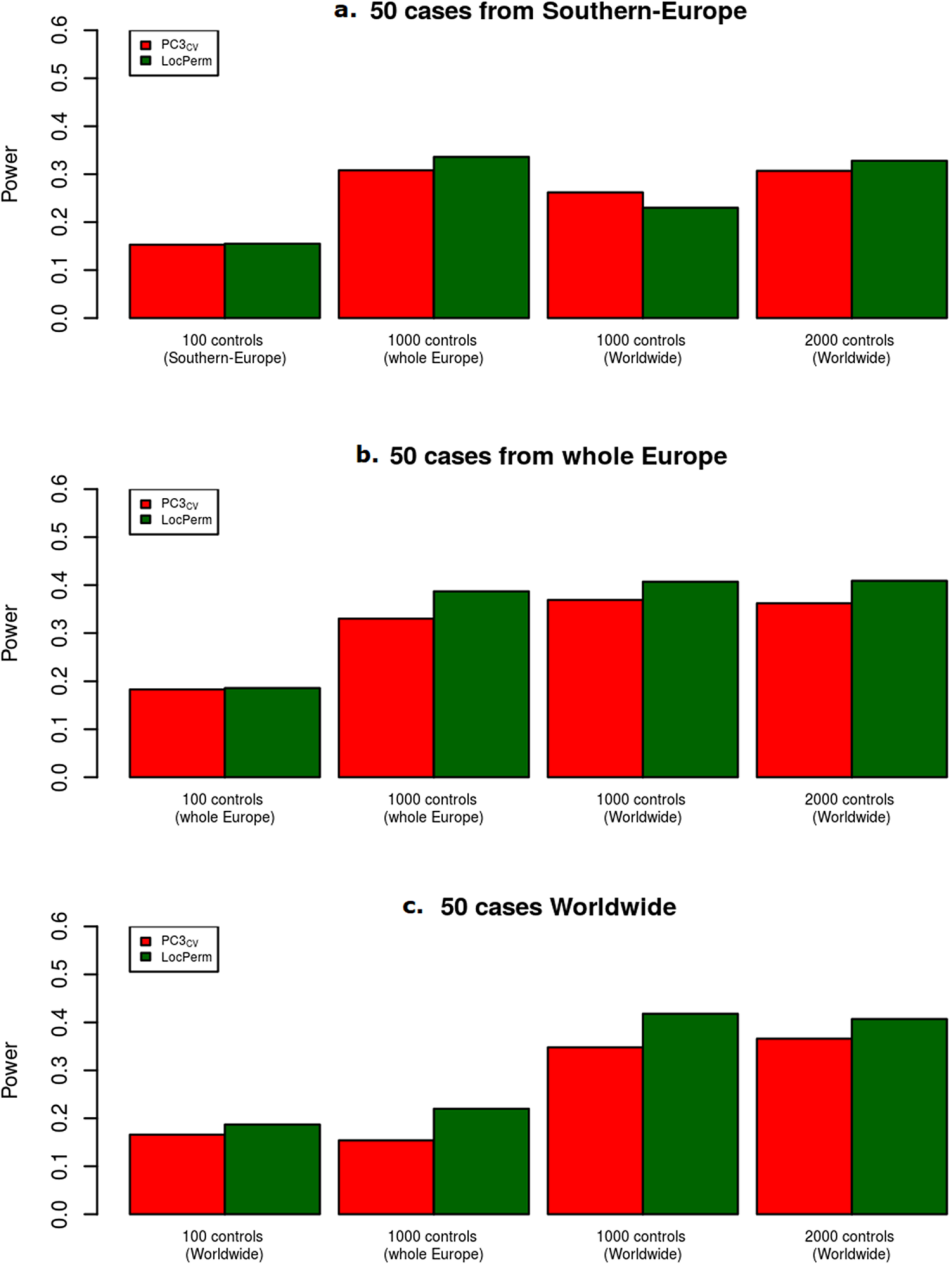
Power for methods with a correct type I error rate under H_0_ for the small size sample at the level $\alpha = 0.001$. (**a**) Scenarios with 50 cases from Southern-Europe. (**b**)Scenarios with 50 cases from the whole Europe. (**c**) Scenarios with 50 cases from the Worldwide sample. The relative risk is fixed at 4.

A power study was performed for PC3_CV_ and LocPerm with small numbers of cases (Fig. 5). Both approaches gave a correct type I error rate and similar results, but power was slightly higher for LocPerm than for PC3 when the 50 cases came from Europe as a whole or from the Worldwide sample. When cases were from Southern Europe, considering 1000 controls from the whole of Europe gave a power twice that obtained when considering 100 controls of the same origin as the cases (Fig. 5a). For example, for a RR of 4 and at α = 0.001, the power increased from 15 to 34% under these conditions with LocPerm. A smaller increase was observed if 1000 controls from the Worldwide sample were used, increasing to a similar level with the use of 2000 Worldwide controls. When the cases were from anywhere in Europe, a similar increase in power was observed with 1000 European and with 1000 Worldwide controls, whereas the use of 2000 Worldwide resulted in no greater power than the use of 1000 Worldwide controls. Finally, when the cases were selected from the Worldwide sample, the use of 1000 Worldwide controls gave a power almost double that achieved with 100 Worldwide controls, whereas the use of 1000 controls from Europe did not substantially increase the power. These results indicated that using a large panel of worldwide controls to increase sample size is a good strategy for increasing the power of a study while correcting for stratification with approaches such as PC3_CV_ or LocPerm.

### Computational considerations

We also assessed the computing time required for the different approaches. While the unadjusted CAST method does not imply the computation of any particular matrix, the same covariance matrix is necessary for PC3_CV_, LMM and LocPerm and additional specific permutation matrices are required for LocPerm only. We ran each method separately, CAST, PC3_CV_ and LocPerm with R, and LMM_CV_ with GEMMA, on the 1,523 individuals and the 17,619 genes of the European sample, under a hypothesis of no association. We broke down the runtime of each method into a pretreatment phase (covariance and permutation matrices) and a gene-testing phase (see Supplementary Table S14 online). The pretreatment runtime was dependent only on the number of individuals (and the set of SNPs used for the calculations) and this part of the analysis was performed only once. The runtime of the gene-testing phase depended on the number of individuals and the number of genes tested, and could be repeated for different analyses (*e.g.* for different MAF thresholds). PC3_CV_ and LMM_CV_ had similar pretreatment times, markedly shorter than that for LocPerm, which also requires the calculation of permutation matrices. However, the need to calculate these matrices only once decreases the relative disadvantage of the LocPerm method. In terms of gene-testing time, LMM_CV_ was the fastest approach when used with GEMMA, but this may not be the case for other programs that have not been optimized. A comparison of the methods implemented with R showed that the adjustment on PCs and LocPerm took 1.4 × and 2.5 × longer, respectively, than the unadjusted test. These comparisons were run on a 64-bit Intel Xeon Linux machine with a CPU of 3.70 GHz and 64 GB of RAM.

## Discussion

We performed a large simulation study based on real exomes data to investigate the ability of several approaches (i.e. PCs, LMM and LocPerm) to account for population stratification in rare-variant association studies of a binary trait. In our simulation study, the efficiency of PCs and LMM to correct for population stratification was dependent on the type of variant used to derive the similarity matrices, the best performance being obtained with CVs. It was generally not possible to correct the stratification bias with RVs, even with the exclusion of private variants for the calculation of the matrices. Private variants have very sparse distributions, which may lead to difficulties in calculation, and their inclusion resulted in an even lower efficiency of correction for population structure (data not shown). Other studies evaluating different types of variants reached the same conclusions^25,26^ although one reported better performances for PC based on RVs^15^. However, this study was based on simulated NGS data, which may have led to an unrealistic rare variant distribution. Our results also indicate that CVs or ALLVs were the best sets of variants for the LMM approach applied to CAST, confirming the results of Luo et al. based on the SKAT test^20^. Variant selection remains an area in which there are perspectives for improving the corrections provided by strategies such as PC or LMM^13,27^, although the use of CVs appeared to be a good choice in most situations.

With the optimal set of variants, PC generally corrected for population stratification more efficiently than LMM. This is consistent with benefits of the PC approach over LMM observed in the presence of spatially confined confounders^45^, which is often the case with rare variants. For large sample sizes, both PC and LMM controlled for stratification better at larger geographic scales than at finer scales. In small samples (50 cases and 100 controls), PC approaches gave inflated type I errors even in the absence of population stratification, as previously reported^19,30,46^. This inflation disappeared when the sample included additional controls, whatever their ethnic origin, even with a highly unbalanced case–control ratio. By contrast, the type I error of LMM was inflated in samples with highly unbalanced case–control ratios, whatever the level of population stratification, as previously noted in the context of GWAS^47^. Finally, the adapted local permutations procedure recently proposed by Mullaert et al.^30^ gave very promising results, as it fully corrected for population stratification, regardless of the scale over which the stratification occurred, sample size and case–control ratio. When valid under H_0_, the three correction methods had similar powers. For a given setting, power was similar in the different stratification situations, indicating that the correction method could maintain the power it would have in the absence of stratification. These results are in partial agreement with several studies reporting a small loss of power for PC-adjusted logistic regression in the presence of stratification relative to an absence of stratification^14,21^.

We also investigated the specific situation in which only a very small number of cases are available, which is particularly relevant in the context of rare disorders. In this setting, we showed that PC and LocPerm provided correct type I errors when the number of controls was large, regardless of the ethnic origin of the controls. In addition, the strategy of adding controls, even of worldwide origin, provided a substantial gain of power for PC and LocPerm when few cases were available. This is an important finding, highlighting the potential interest of using publicly available controls, such as those of the 1000G project, to increase the power of a study with a small sample size. We also investigated an additional scenario in which all cases were strictly from our in-house HGID cohort and the controls were obtained from both the HGID and 1000 Genomes cohorts (data not shown). This scenario gave identical results to those presented here, indicating that, even in the presence of heterogeneity in the types of exome data considered for cases and controls (*e.g.* in terms of kit or technology used), the conclusions drawn here still apply. Overall, these results validate a strategy of using additional external controls to increase the power of a study, provided that an efficient stratification correction approach is used.

We focused on the investigation of rare diseases caused by a few deleterious variants, for which the CAST-like approach is particularly appropriate. Additional studies are required to investigate more complex genetic models, such as the presence of both risk and protective variants of a given gene, for which other association tests, such as variant-component approaches, may be more appropriate. Different results can be expected, as the effect of population stratification differs between testing strategies^18,21^. In the situations we considered, our study highlighted several useful conclusions for rare variant association studies in the presence of stratification: (1) the key issue is to properly control type I errors as powers are comparable, (2) population stratification can be corrected by PC3_CV_ in most instances, unless there is a high degree of intracontinental stratification and a small sample size, (3) LocPerm proposes a satisfying correction in all instances, and (4) strategies based on the inclusion of a large number of additional controls (*e.g.* from publicly available databases) provide a substantial gain of power provided that stratification is controlled for correctly.

## Supplementary Information

Supplementary Information 1.

## Data Availability

The datasets generated during and/or analysed during the current study are available from the corresponding author upon reasonable request.
