# Supplementary material for: Controlling for human population stratification in rare variant association studies
Source: Sci Rep. 2021 Sep 24;11:19015. doi: 10.1038/s41598-021-98370-5 (PMC8463695; doi:10.1038/s41598-021-98370-5)
Supplement: Supplementary file 1 — Supplementary Information 1. [file 41598_2021_98370_MOESM1_ESM.pdf]

Controlling for Human Population Stratification in Rare Variant Association Studies

Matthieu Bouaziz[1,2], Jimmy Mullaert[1,2,3,4], Benedetta Bigio[5], Yoann Seeleuthner[1,2], Jean-Laurent Casanova[1,2,5,6,7], Alexandre Alcais[1,2], Laurent Abel[1,2,5, ¶], Aurélie Cobat[1,2, ¶,*] .

1.Laboratory of Human Genetics of Infectious Diseases, Necker Branch, INSERM U1163, Paris, France, EU.

2. Université de Paris, Imagine Institute, 75015 Paris, France, EU.

3. Université de Paris, IAME, INSERM, F-75018 Paris, France

4. AP-HP, Hôpital Bichat, DEBRC, F-75018 Paris, France

5. St. Giles Laboratory of Human Genetics of Infectious Diseases, Rockefeller Branch, The Rockefeller University, New York, USA.

6. Howard Hughes Medical Institute, New-York, NY, USA

7. Pediatric Hematology and Immunology Unit, Necker Hospital for Sick Children, 75015 Paris, France, EU.

[¶] These authors contributed equally to this work

* Corresponding author : aurelie.cobat@inserm.fr (AC)

Supplementary material

This file contains 14 supplementary tables and 5 supplementary figures. Legends for these elements are provided in the main article.

Supplementary Table S1. Distribution of the samples in the European and Worldwide sub-populations

European cohort				
**Group**	**Northern-Europe**	**Middle-Europe**		**Sourthern-Europe**
**#Samples**	127	651		745
**Worldwide cohort**				
**Group**	**Europe**	**South-Asia**	**North-Africa**	**Middle-East**
**#Samples**	700	543	359	365

Supplementary Table S2. Distribution of the variants in the European and the Worldwide samples according to their MAFs as described in the Material and Methods section.

**Type of variants**	**MAFs**	**European cohort**	**Worldwide cohort**
Private	Singleton	102,219	132,565
Rare	$0\% < MAF < 1\%$	186,991	332,850
Low-frequency	$1\% \leqslant MAF < 5\%$	48,167	53,940
Common	$MAF \geqslant 5\%$	93,831	96,972
All	All but private	328,989	483,762

Supplementary Table S3: Distribution of the cases in the sub-populations of the European and the Worldwide cohorts for the different population stratification (PS) scenarios.

[a] #cases (% of the sub-population)

European cohort				
**Scenario**	**Northern-Europe (n=127)**	**Middle-Europe (n=651)**		**Southern-Europe (n=745)**
**No PS**	19 (15 %)[a]	98 (15 %)		112 (15 %)
**Moderate PS**	6 (5 %)	45 (7 %)		177 (24 %)
**High PS**	0 (0 %)	0 (0 %)		228 (30 %)
**Worldwide cohort**				
**Scenario**	**Europe (n=700)**	**South-Asia (n=543)**	**North-Africa (n=359)**	**Middle-East (n=365)**
**No PS**	105 (15 %)[a]	81 (15 %)	53 (15 %)	54 (15 %)
**Moderate PS**	177 (25 %)	60 (11 %)	29 (8 %)	29 (7 %)
**High PS**	294 (42 %)	0 (0 %)	0 (0 %)	0 (0 %)

Supplementary Table S4: Stratification scenarios for the small size study. The first 4 scenarios correspond to cases from the Southern-Europe sub-population (SE), the following 4 scenarios to cases from whole European sample (E) and the final 4 to cases from the Worldwide population (W). Controls are randomly drawn among the Southern-European, European or Worldwide populations.

Scenario	Cases	Controls
**50SE-100SE**	50 from Southern-Europe	100 from Southern-Europe
**50SE-1000E**	50 from Southern-Europe	1000 from all Europe
**50SE-1000W**	50 from Southern-Europe	1000 Worldwide
**50SE-2000W**	50 from Southern-Europe	2000 Worldwide
**50E-100E**	50 from all Europe	100 from all Europe
**50E-1000E**	50 from all Europe	1000 from all Europe
**50E-1000W**	50 from all Europe	1000 Worldwide
**50E-2000W**	50 from all Europe	2000 Worldwide
**50W-100W**	50 Worldwide	100 Worldwide
**50W-1000E**	50 Worldwide	1000 from all Europe
**50W-1000W**	50 Worldwide	1000 Worldwide
**50W-2000W**	50 Worldwide	2000 Worldwide

Supplementary Table S5. Number of genes tested and 95%PI in each scenario of the small sample study. Prediction intervals are adjusted on the 4 methods tested.

Scenario	Average # genes per replicate	IC95 % - $\alpha = 0.001$	IC95 % - $\alpha = 0.01$
**50SE-100SE**	12,695	0.00078 – 0.00122	0.00930 – 0.01070
**50SE-1000E**	17,246	0.00081 – 0.00119	0.00940 – 0.01060
**50SE-1000W**	17,900	0.00081 – 0.00119	0.00941 – 0.01059
**50SE-2000W**	18,111	0.00081 – 0.00119	0.00942 – 0.01058
**50E-100E**	12,630	0.00078 – 0.00122	0.00930 – 0.01070
**50E-1000E**	17,240	0.00081 – 0.00119	0.00940 – 0.01060
**50E-1000W**	17,954	0.00081 – 0.00119	0.00941 – 0.01059
**50E-2000W**	18,109	0.00081 – 0.00119	0.00942 – 0.01058
**50W-100W**	13,761	0.00079 – 0.00121	0.00933 – 0.01067
**50W-1000E**	17,543	0.00081 – 0.00119	0.00941 – 0.01059
**50W-1000W**	17,956	0.00081 – 0.00119	0.00941 – 0.01059
**50W-2000W**	18,187	0.00081 – 0.00119	0.00942 – 0.01058

Supplementary Table S6. Details of the genes selected for the power analysis in the European and the Worldwide samples. Freq() indicates the cumulative frequency of the causal variants.

Gene	Chr	#Variants	#RVs	No Causal RVs	Freq(Causal RVs) in Europe	Freq(Causal RVs) in Worldwide
ADAMTS4	1	32	25	12	0.043	0.038
ZSWIM5	1	32	28	14	0.041	0.053
TDRD15	2	32	31	14	0.028	0.031
GTF2E1	3	27	26	11	0.035	0.024
ALAD	9	32	23	12	0.049	0.042
RP11-248J23.7	10	29	25	12	0.041	0.037
STOML3	13	25	20	10	0.038	0.034
OR4N2	14	42	34	16	0.061	0.067
NLRP4	19	49	40	17	0.057	0.046
CSRP2BP	20	39	30	12	0.021	0.021

Supplementary Table S7. Example of the penetrance parameters used for the power simulation study of the gene ADAMTS4 in the European sample. Penetrance (i.e. probabilities of the disease) for non-carriers (F_0) and carriers of at least one causal variant (F_1) were estimated in each European subgroup according the population stratification scenario and for a relative risk RR of 3 using the following formulas: $P(D) = F_0.P(G_0) + F_1.P(G_1) \Leftrightarrow F_0 = P(D) / (P(G_0) + RR.P(G_1))$, and $F_1 = RR.F_0$, where P(D) is the disease frequency in a given subgroup group and varies according to the population stratification scenario, $P(G_1)$ is the frequency of carriers observed in the subgroup and $P(G_0)$ the frequency of non-carriers.

	**No PS**			**Moderate PS**			**High PS**		
	**NE**	**ME**	**SE**	**NE**	**ME**	**SE**	**NE**	**ME**	**SE**
**P(D)**	0.15	0.15	0.15	0.05	0.07	0.24	0	0	0.30
**$P(G_1)$**	0.09	0.09	0.08	0.09	0.09	0.08	0.09	0.09	0.08
**$P(G_0)$**	0.91	0.91	0.92	0.91	0.91	0.92	0.91	0.91	0.92
**F_0**	0.13	0.13	0.13	0.040	0.060	0.210	0.000	0.000	0.260
**F_1**	0.39	0.39	0.39	0.120	0.180	0.630	0.000	0.000	0.780

Supplementary Table S8. Type I error rates of the different approaches for the large European sample. The nominal level alpha considered is $\alpha = 0.01$ and the corresponding 95%PI adjusted for the 10 methods is [0.00933-0.01067]. Type I error rates under the lower bound of the 95%PI are displayed in italic and above the upper bound of the 95%PI in bold.

**A - No Stratification**				
	**CAST**	**PC3**	**LMM**	**LocPerm**
**RVs**	0.01045	**0.0107**	0.00981	0.00972
**LFVs**		0.0106	0.00975	
**CVs**		**0.01072**	0.00978	
**ALLVs**		0.01065	0.0098	
**B – Moderate Stratification**				
	**CAST**	**PC3**	**LMM**	**LocPerm**
**RVs**	**0.0144**	**0.01128**	0.01055	0.00946
**LFVs**		**0.01102**	0.0106	
**CVs**		0.01042	0.0106	
**ALLVs**		0.0106	0.00992	
**C – High Stratification**				
	**CAST**	**PC3**	**LMM**	**LocPerm**
**RVs**	**0.02258**	**0.01271**	**0.01162**	*0.00863*
**LFVs**		**0.01175**	**0.01201**	
**CVs**		**0.01271**	**0.01176**	
**ALLVs**		**0.01159**	0.01037	

Supplementary Table S9. Type I error rates of the PC approach with 3, 5, 10 or 50 PCs for the large European sample. The nominal level alpha considered is $\alpha = 0.001$ and the corresponding 95%PI adjusted for the 16 methods is [0.00078-0.00122]. Type I error rates under the lower bound of the 95%PI are displayed in italic and above the upper bound of the 95%PI in bold.

**A - No Stratification**				
	**PC3**	**PC5**	**PC10**	**PC50**
**RVs**	0.00108	0.00111	0.00111	**0.00141**
**LFVs**	0.0011	0.00111	0.00104	**0.00127**
**CVs**	0.00104	0.00106	0.00104	**0.00125**
**ALLVs**	0.00108	0.00108	0.00108	**0.00138**
**B – Moderate Stratification**				
	**PC3**	**PC5**	**PC10**	**PC50**
**RVs**	0.00117	0.00110	**0.00124**	**0.00159**
**LFVs**	0.00101	0.00108	0.00111	**0.00124**
**CVs**	0.001	0.00111	0.00108	**0.00125**
**ALLVs**	0.00102	0.00106	0.00116	**0.00152**
**C – High Stratification**				
	**PC3**	**PC5**	**PC10**	**PC50**
**RVs**	**0.00157**	**0.00160**	**0.00138**	**0.00188**
**LFVs**	**0.00137**	**0.00130**	**0.00131**	**0.00160**
**CVs**	**0.00136**	**0.00124**	**0.00126**	**0.00147**
**ALLVs**	**0.00133**	**0.00130**	**0.00140**	**0.00181**

Supplementary Table S10. Type I error rates of WSS and SKAT approaches for the large European sample. The nominal level alpha considered is $\alpha = 0.001$ and the corresponding 95%PI adjusted for the 10 methods is [0,00079-0,00121]. Type I error rates under the lower bound of the 95%PI are displayed in italic and above the upper bound of the 95%PI in bold.

A - No Stratification

	**CAST**	**CAST-PC3**	**CAST-LMM**	**CAST-LocPerm**	**WSS**	**WSS-PC3**	**SKAT**	**SKAT-PC3**
CVs	0.00106	0.00104	0.00118	0.00082	0.0011	0.0011	0.00117	0.00114

B – Moderate Stratification

	**CAST**	**CAST-PC3**	**CAST-LMM**	**CAST-LocPerm**	**WSS**	**WSS-PC3**	**SKAT**	**SKAT-PC3**
CVs	**0.00163**	0.001	**0.00124**	0.00095	**0.0018**	0.00107	**0.0024**	**0.00126**

C – High Stratification

	**CAST**	**CAST-PC3**	**CAST-LMM**	**CAST-LocPerm**	**WSS**	**WSS-PC3**	**SKAT**	**SKAT-PC3**
CVs	**0.00359**	**0.00136**	**0.00161**	0.00087	**0.0039**	**0.0017**	**0.0053**	**0.00129**

Supplementary Table S11. Type I error rates of the different approaches for the large Worldwide sample. The nominal level alpha considered is $\alpha = 0.01$ and the corresponding 95%PI adjusted for the 10 methods is [0.00933-0.01067]. Type I error rates under the lower bound of the 95%PI are displayed in italic and above the upper bound of the 95%PI in bold.

**A - No Stratification**				
	**CAST**	**PC3**	**LMM**	**LocPerm**
**RVs**	0.00955	0.01005	*0.00921*	0.00958
**LFVs**		0.01046	*0.00918*	
**CVs**		0.01027	*0.00932*	
**ALLVs**		0.01042	*0.00921*	
**B – Moderate Stratification**				
	**CAST**	**PC3**	**LMM**	**LocPerm**
**RVs**	**0.03597**	**0.01676**	**0.02824**	0.00985
**LFVs**		**0.01171**	0.00965	
**CVs**		**0.01099**	*0.00886*	
**ALLVs**		**0.01172**	**0.01325**	
**C – High Stratification**				
	**CAST**	**PC3**	**LMM**	**LocPerm**
**RVs**	**0.24837**	**0.03309**	*0.06683*	0.00959
**LFVs**		**0.01219**	**0.01107**	
**CVs**		**0.01148**	*0.00896*	
**ALLVs**		**0.0115**	**0.01702**	

Supplementary Table S12. Type I error rates of the PC approach with 3, 5, 10 or 50 PCs for the large Worldwide sample. The nominal level alpha considered is $\alpha = 0.001$ and the corresponding 95%PI adjusted for the 16 methods is [0.00078-0.00122]. Type I error rates under the lower bound of the 95%PI are displayed in italic and above the upper bound of the 95%PI in bold.

**A - No Stratification**				
	**PC3**	**PC5**	**PC10**	**PC50**
**RVs**	0.00099	0.00102	0.00102	**0.00129**
**LFVs**	0.00099	0.00096	0.00097	0.00108
**CVs**	0.00099	0.00099	0.001	0.0012
**ALLVs**	0.00099	0.00098	0.00101	**0.00131**
**B – Moderate Stratification**				
	**PC3**	**PC5**	**PC10**	**PC50**
**RVs**	**0.00259**	**0.00263**	**0.00267**	**0.00284**
**LFVs**	0.00109	0.00119	0.0011	**0.00132**
**CVs**	0.00105	0.00113	0.00115	**0.00132**
**ALLVs**	**0.00128**	**0.00131**	**0.00135**	**0.00149**
**C – High Stratification**				
	**PC3**	**PC5**	**PC10**	**PC50**
**RVs**	**0.00662**	**0.00658**	**0.00661**	**0.00761**
**LFVs**	0.0012	0.00119	0.00117	**0.00165**
**CVs**	0.00119	**0.00124**	**0.00125**	0.00116
**ALLVs**	**0.00127**	**0.00225**	**0.00201**	**0.00212**

Supplementary Table S13. Type I error rates of the different approaches for the small sample scenarios. The nominal level alpha considered is $\alpha = 0.01$. Type I error rates under the lower bound of the 95%PI are displayed in italic and above the upper bound of the 95%PI in bold.
Table S3 provides the adjusted 95%PI for the different number of genes tested in each scenario.

Scenario	CAST	$PC3_{CV}$	LMM_{CV}	LocPerm
**50SE-100SE**	**0.01150**	**0.01260**	0.01060	0.01022
**50SE-1000E**	**0.01340**	**0.01140**	**0.01280**	*0.00895*
**50SE-1000W**	**0.02772**	**0.01153**	*0.00891*	*0.00932*
**50SE-2000W**	**0.02870**	**0.01120**	*0.00920*	*0.00937*
**50E-100E**	**0.01100**	**0.01190**	0.01040	0.00999
**50E-1000E**	0.01010	0.01030	**0.01080**	*0.00885*
**50E-1000W**	**0.03090**	**0.01081**	*0.00831*	*0.00928*
**50E-2000W**	**0.03060**	**0.01060**	*0.00860*	0.00963
**50W-100W**	**0.0121**	**0.0126**	**0.0108**	0.01036
**50W-1000E**	**0.0337**	*0.0082*	**0.0204**	0.00940
**50W-1000W**	0.01053	**0.0109**	**0.0109**	0.00959
**50W-2000W**	0.0104	**0.0106**	**0.0108**	*0.00926*

Supplementary Table S14. Runtime of each method calculated on 1,523 individuals and 17,619 genes of the large European sample under the null hypothesis. Note that if the analyses are conducted several times, with for instance different MAF thresholds or modes of inheritance, the pre-treatment part does not have to be performed again.

Method	Pre-treatment (sec)	Gene-testing (sec)	Total (sec)
**CAST**	0	196	196
**CAST-PC**	60	268	328
**LMM**	60	68	128
**Adapted Permutation**	2880	500	3380

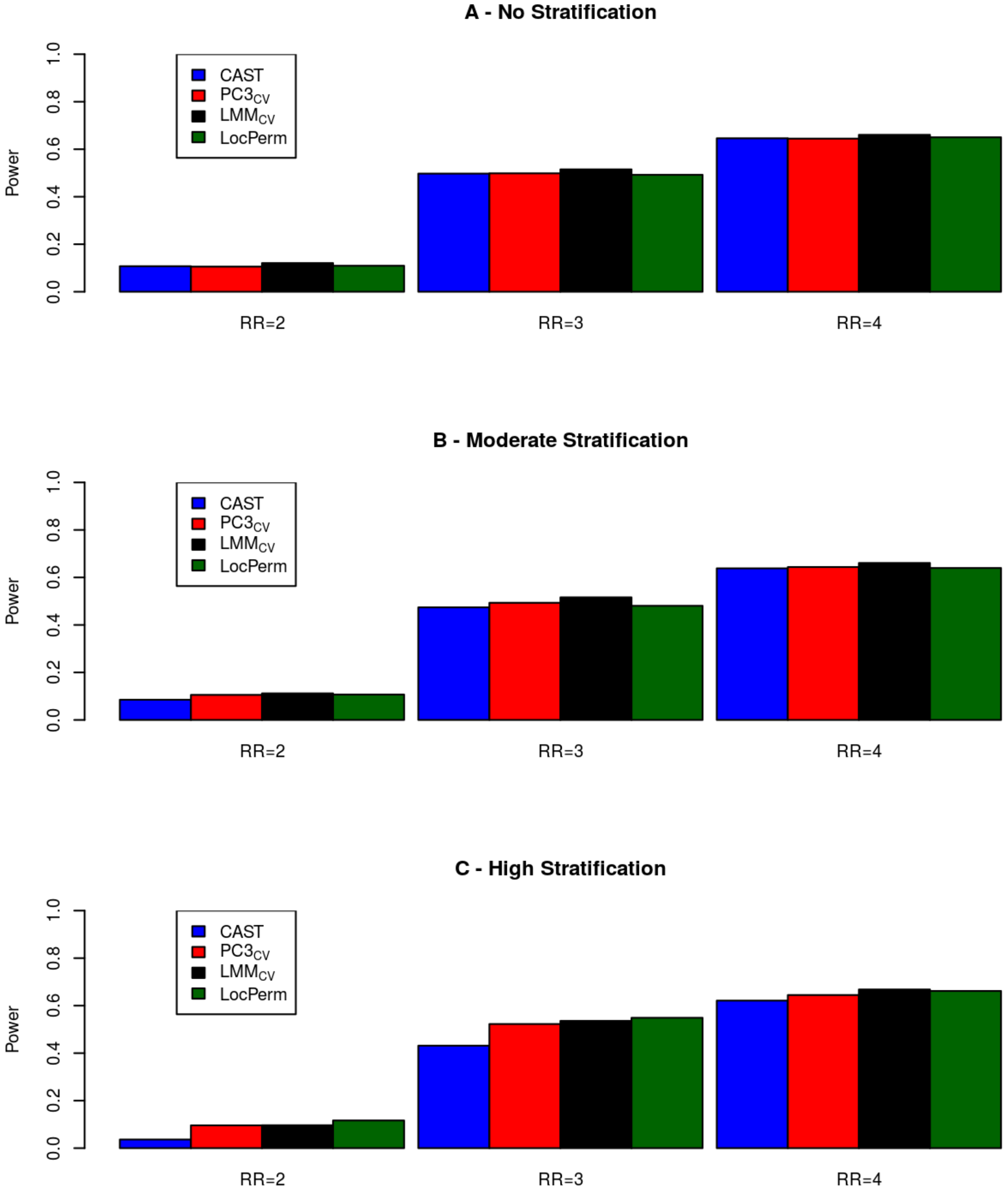

Supplementary Figure S1. Histogram of adjusted powers of the correction methods for the large European sample (n=1,523) at the level $\alpha = 0.001$. (A) Without stratification. (B) With moderate stratification. (C) With high stratification. Relative risks considered vary from 2 to 4 on the x-axis.

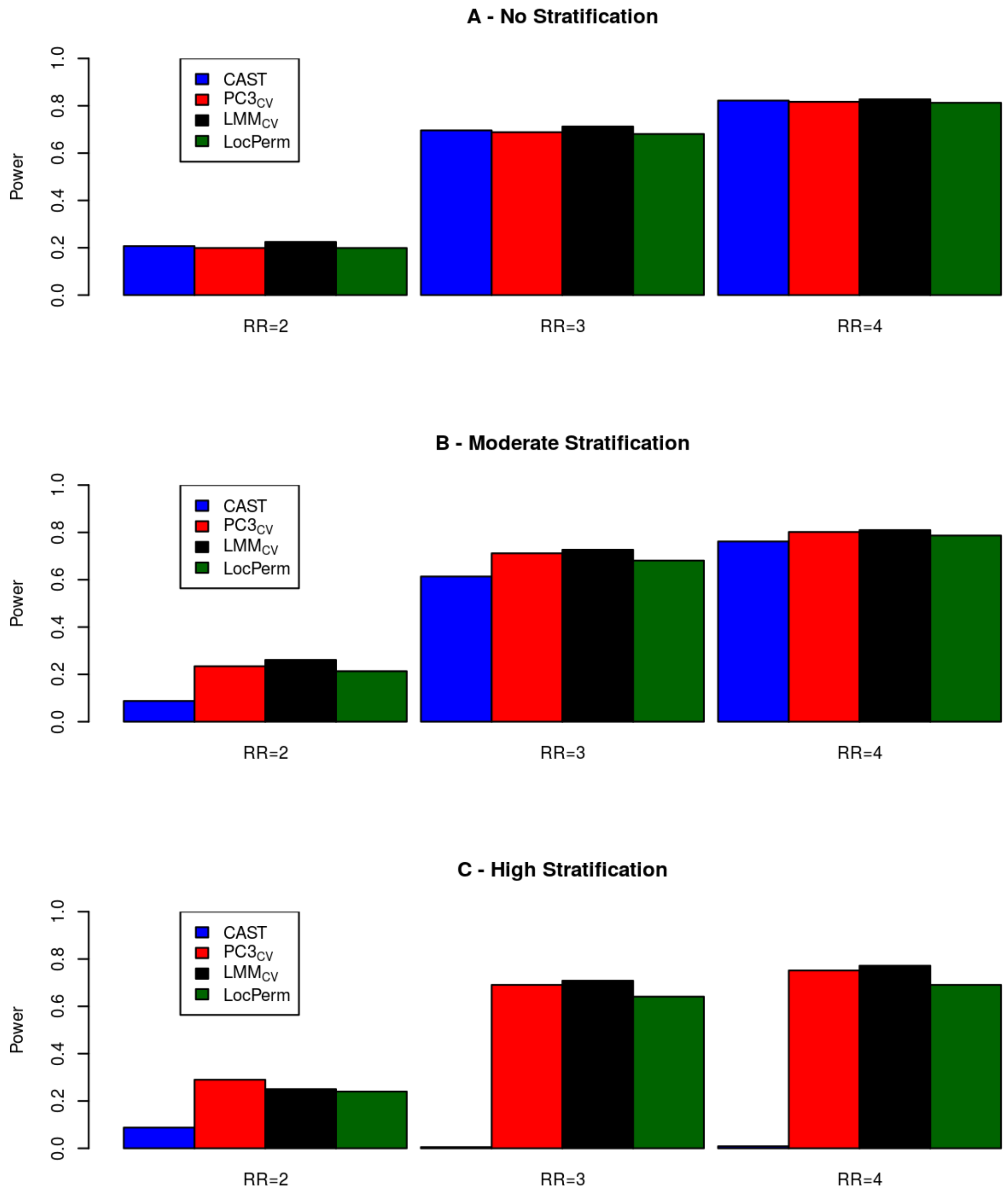

Supplementary Figure S2. Histogram of adjusted powers for the correction methods for the large Worldwide sample at the level $\alpha = 0.001$. (A) Without stratification. (B) With moderate stratification. (C) With high stratification. Relative risks considered vary from 2 to 4 on the x-axis.

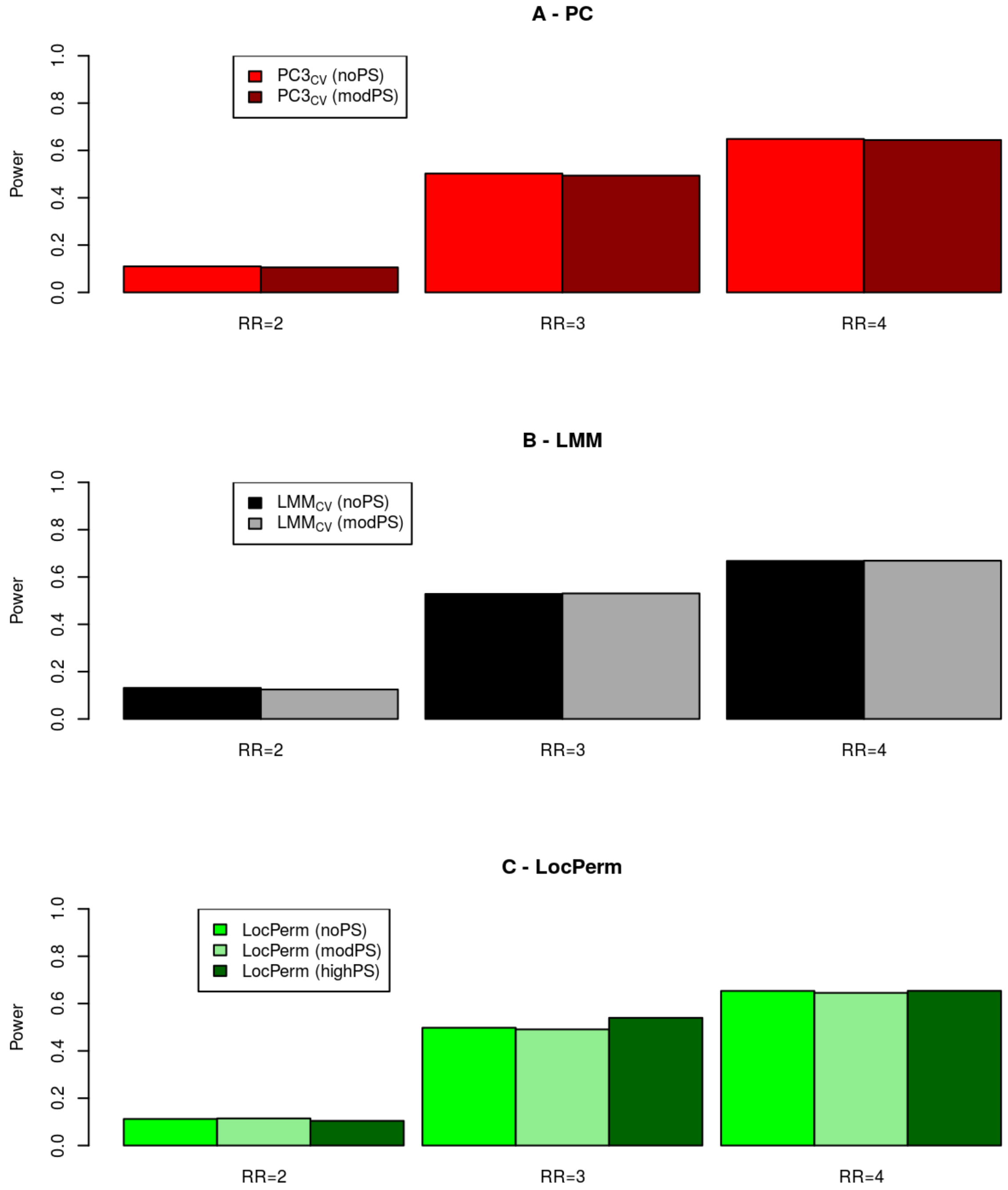

Supplementary Figure S3. Histogram of powers for methods with a correct type I error rate for the large European sample (n=1,523) at the level $\alpha = 0.001$. (A) Principal components. (B) Linear Mixed Models. (C) LocPerm. Relative risks vary from 2 to 4 on the x-axis.

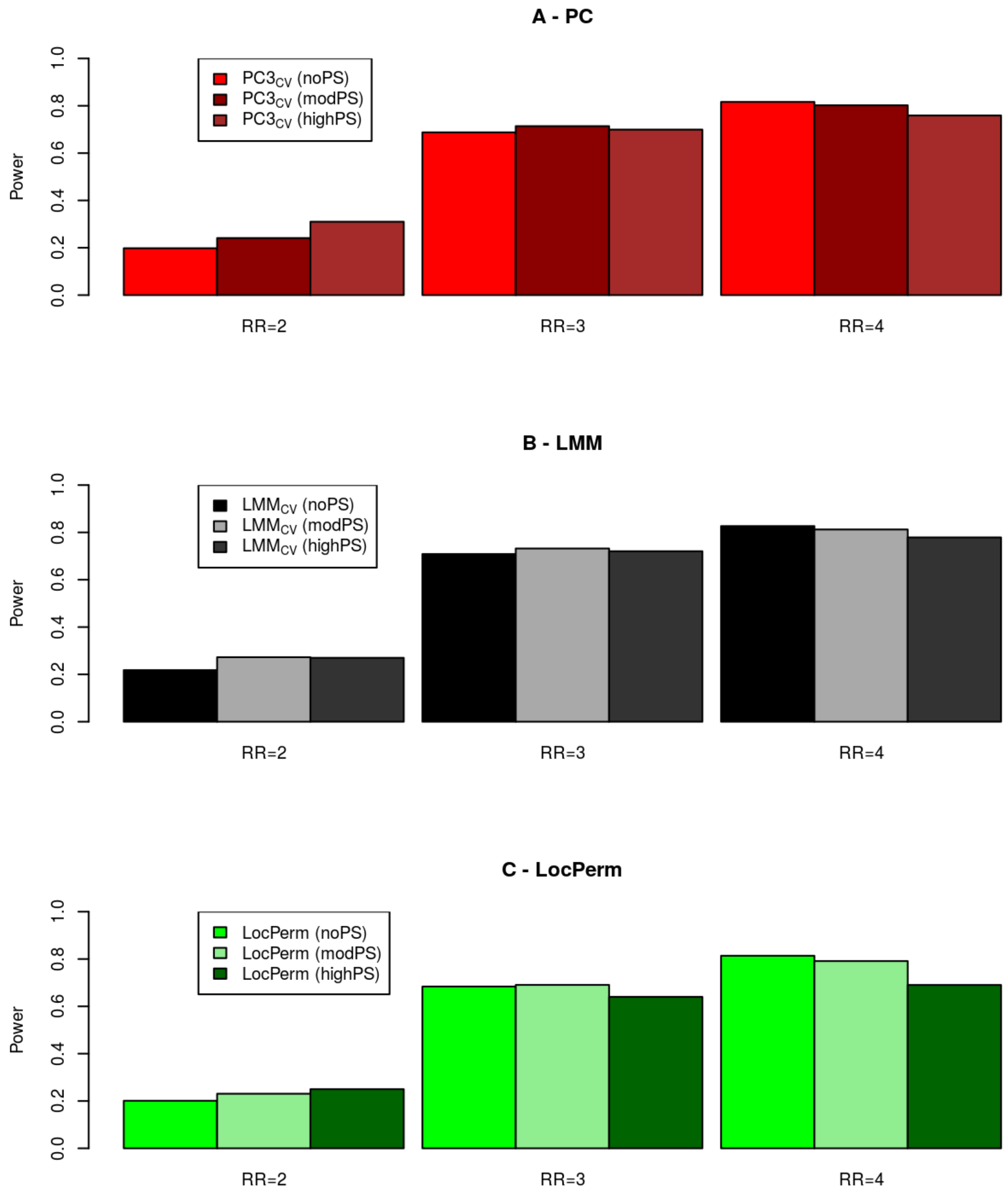

Supplementary Figure S4. Histogram of powers for methods with a correct type I error rate for the large Worldwide sample at the level $\alpha = 0.001$. (A) Principal components. (B) Linear Mixed Models. (C) LocPerm. Relative risks vary from 2 to 4 on the x-axis.

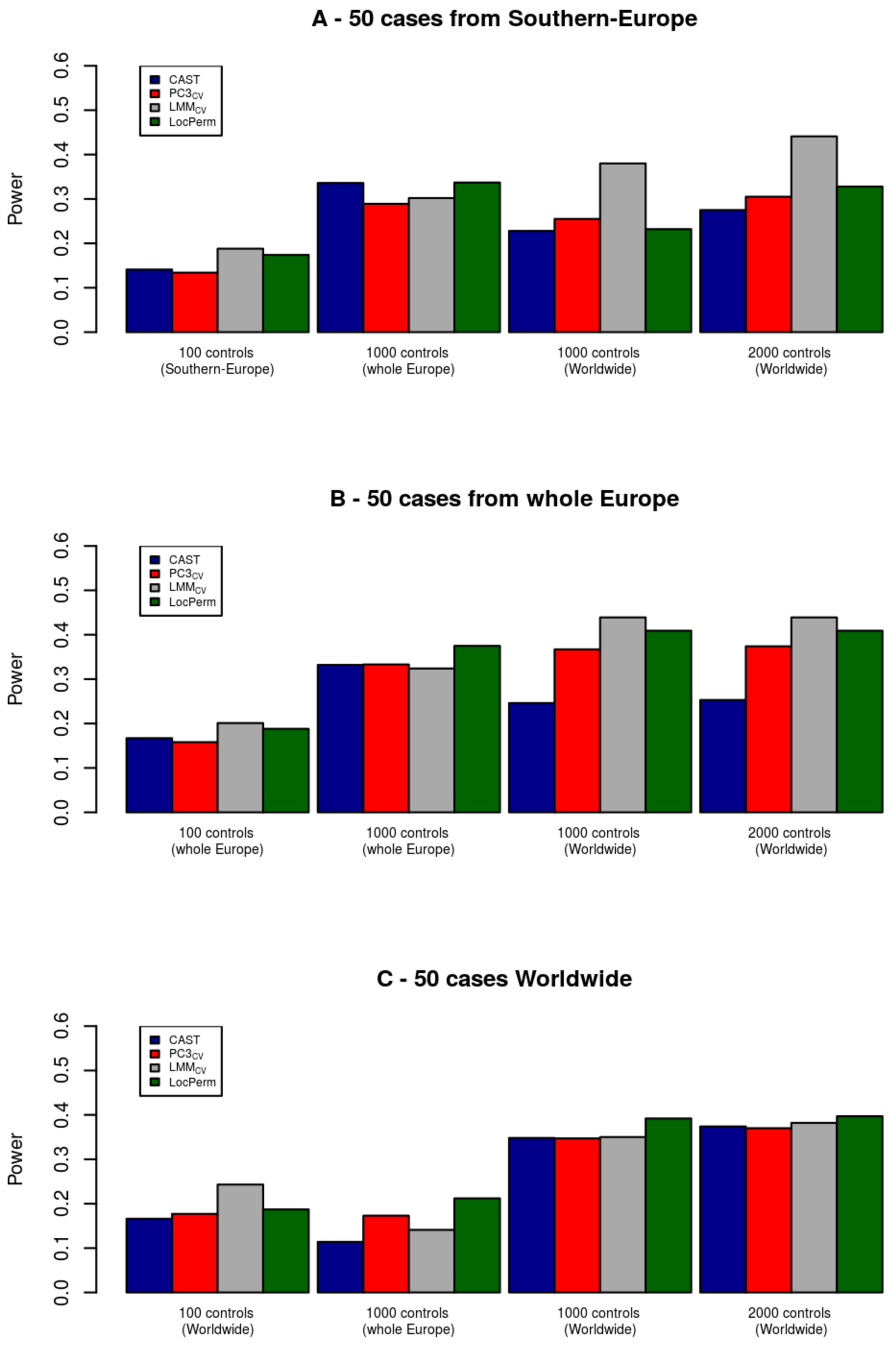

Supplementary Figure S5. Histogram of adjusted powers of the correction methods the small sample at the level $\alpha = 0.001$. (A) Scenarios with 50 cases from Southern-Europe. (B)Scenarios with 50 cases from the whole Europe. (C) Scenarios with 50 cases from the Worldwide sample. The relative risk is fixed at 4.
